# Divalent cation-induced conformational changes of influenza virus hemagglutinin

**DOI:** 10.1038/s41598-020-72368-x

**Published:** 2020-09-22

**Authors:** Jong Hyeon Seok, Hyojin Kim, Dan Bi Lee, Jeong Suk An, Eun Jeong Kim, Ji-Hye Lee, Mi Sook Chung, Kyung Hyun Kim

**Affiliations:** 1 Department of Biotechnology and Bioinformatics, Korea University, Sejong, 30019 Korea; 2 Department of Food and Nutrition, Duksung Women’s University, Seoul, 01369 Korea

**Keywords:** Influenza virus, X-ray crystallography

## Abstract

Divalent cations Cu^2+^ and Zn^2+^ can prevent the viral growth in mammalian cells during influenza infection, and viral titers decrease significantly on a copper surface. The underlying mechanisms include DNA damage by radicals, modulation of viral protease, M1 or neuraminidase, and morphological changes in viral particles. However, the molecular mechanisms underlying divalent cation-mediated antiviral activities are unclear. An unexpected observation of this study was that a Zn^2+^ ion is bound by Glu68 and His137 residues at the head regions of two neighboring trimers in the crystal structure of hemagglutinin (HA) derived from A/Thailand/CU44/2006. The binding of Zn^2+^ at high concentrations induced multimerization of HA and decreased its acid stability. The acid-induced conformational change of HA occurred even at neutral pH in the presence of Zn^2+^. The fusion of viral and host endosomal membranes requires substantial conformational changes in HA upon exposure to acidic pH. Therefore, our results suggest that binding of Zn^2+^ may facilitate the conformational changes of HA, analogous to that induced by acidic pH.

## Introduction

Influenza viruses belonging to the family *Orthomyxoviridae* comprise four antigenic types: A, B, C, and, D. Influenza A and B viruses co-circulate and cause seasonal epidemics, and influenza C virus causes severe disease in children^1,2^. Based on the antigenicity of hemagglutinin (HA) and neuraminidase (NA), 18 HA and 11 NA subtypes of influenza A viruses have been identified in wild waterfowl, and subtypes H17N10 and H18N11 have been discovered in bats ^3,4^. The various subtypes are divided into two groups, group 1 (including H1, H2 and H5) and group 2 (including H3 and H7). Thus far, three HA (H1, H2, and H3) and two NA (N1 and N2) subtypes have adapted to humans to produce pandemic (pdm) and seasonal influenza A viruses^5^. The dynamic nature of influenza viral infections, particularly due to the variation of HA, remains a big challenge in minimizing the mismatch between circulating seasonal strains and vaccines.

Progression of viral particles from early to late endosomes results in large conformational changes of the active form of HA that is derived from the proteolytic cleavage of HA0 precursor into HA1 and HA2^6,7^. The HA structure has two distinct regions. The head region is composed of the receptor binding and vestigial esterase domains of HA1, whereas the stem region includes a long central helix of HA2. The HA plays a critical role for acidic pH-induced membrane fusion in viral fitness^8,9^ and involves the dissociation of the head regions and the extension of central coiled-coil domains in the stem regions. In this context, the stability of HA is an important attribute of influenza viral fitness, which regulates and enhances viral growth and pathogenesis^10,11^. The stability of HA is changed significantly within and among the subtypes^10,12^, and the recent A(H1N1) 2009pdm viruses evolved to exhibit significantly more stable HA as they became seasonal strains circulating globally^13,14^. Mutations in the coiled coil region of HA2 and at the interface between HA1 and HA2 subunits influence the stability of HA^15–18^. We also demonstrated that the recombinant HA derived from A/Korea/01/2009, a 2009pdm isolate, is a monomer in solution and crystals, where HA molecules are arranged in a head-to-head manner^19^. However, the monomeric HA has a significantly lower stability than the trimer.

The influenza A viruses contain a significant number of Zn^2+^ ion^20^. The Zn^2+^ is shown to bind to M1 protein with a tetrahedral coordination with two Cys and two His residues, where the Zn^2+^ ion may play a structural role^21^. Cu^2+^ and Zn^2+^ ions, albeit less effective in the latter, significantly reduced the influenza viral titers in MDCK cells and on a copper surface^22,23^. Zn^2+^ is also an important cofactor of many viral proteins, including nucleocapsid protein 7 of HIV-1, NS3 of hepatitis C virus, VP30 of Ebola virus, E7 of human papilloma virus, VP6 of rotavirus, and E protein of Semliki Forest virus^24–28^, mainly showing a structural role in zinc binding motifs. However, our understanding of the molecular mechanisms of divalent cation-mediated antiviral activities is far from complete. In this study, solving the crystal structure of HA derived from A/Thailand/CU44/2006 (CU44) led to a surprising finding of a divalent cation Zn^2+^ bound to the head regions of two neighboring trimers. The divalent cation in the crystal structure appeared to enhance the stability of HA, but significantly decreased the melting temperature T_m_ of the CU44 HA, depending on the Zn^2+^ concentrations. The acid-induced conformational change of HA occurred even at neutral pH in the presence of Zn^2+^. Therefore, our results suggest that binding of Zn^2+^ may facilitate the conformational change of HA, analogous to that induced by acidic pH. Here, we report the structure of HA bound to Zn^2+^ ions and the effects of the divalent cation on pH-dependent conformational changes of HA.

## Results

### Structure of Cu44 HA with Zn^2+^ ion

We originally aimed to improve the stability of HA in solution and crystals to allow high resolution analyses of how pH-induced conformational changes affect its biological functions. A total of nine mutants of the CU44 HA wild-type were constructed: L69S, I71S, L156S, I184S, F260A, and A276T of HA1 in the head region, and L73S, M77I, and V91I of HA2 in the stem region (based on H3 numbering). The mutations largely accounted for reduced exposure of hydrophobic amino acid residues on the surface and increased hydrophobic residues at the monomer–monomer interface within the trimer. They were expressed in insect cell culture, purified, and characterized by differential scanning fluorimetry (DSF). No improvement in protein stability was seen in terms of melting temperature (T_m_).

**Figure 1:**
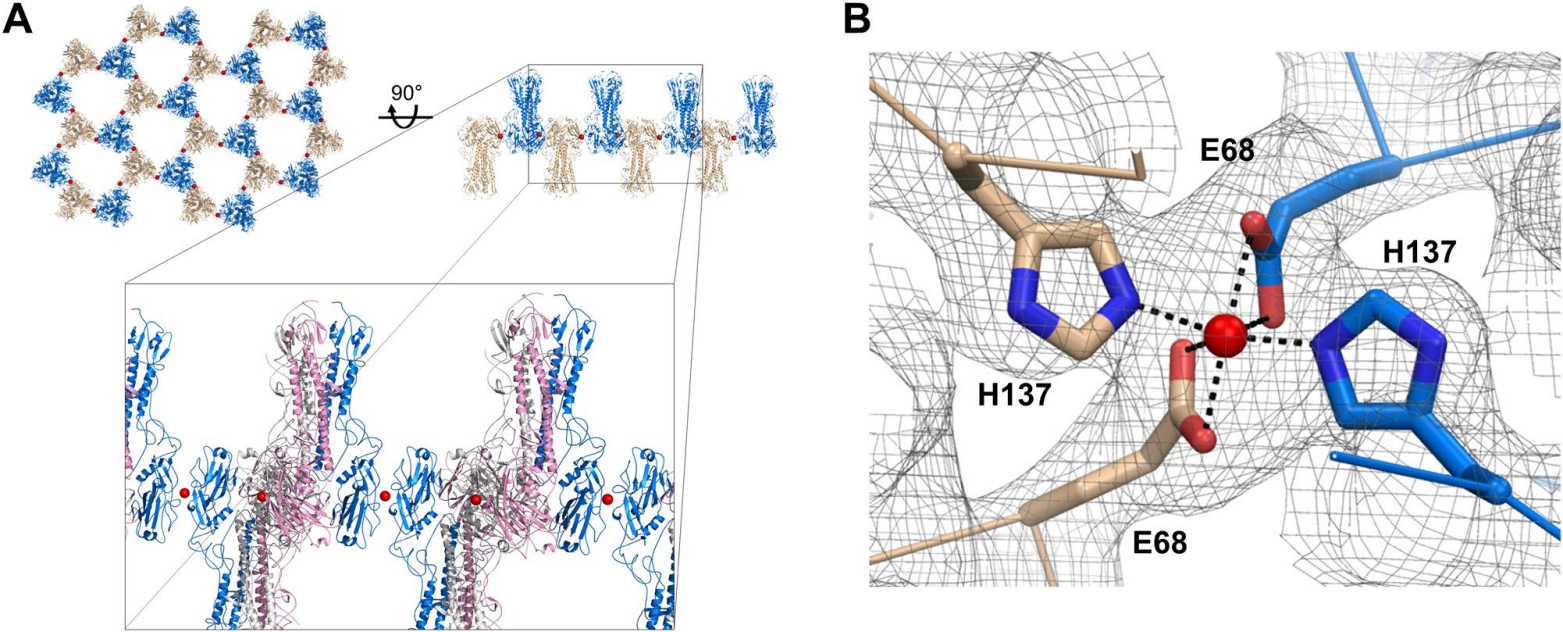
Overview of the structure of the CU44 HA. (**A**) Top and side views along the C axis of the crystal packing of HA. The HA trimers adopt an alternating up and down arrangement with head-to-head interactions, which are shown in different colors. Divalent cations are shown as red spheres. A magnified view of the HA trimers, which are shown in secondary structures in different colors (blue, pink, and lime green) for each monomer. (**B**) Zn^2+^ ion binding site showing an octahedral coordination with two Glu68 and two His137 residues, from two adjacent HA trimers, in the 2|F_o_| − |F_c_| electron density contoured at 1.2 σ. The figures were created with PyMOL (https://www.pymol.org/).

We attempted to determine the crystal structure of the CU44 HA wild type as a control, by molecular replacement using PDB 4EDB as a template. The structure in space group C2 (a = 215.7 Å, b = 124.3 Å, c = 214.5 Å, b = 102.9°) was refined at 3.2 Å resolution with an R/R_free_ of 26.3/29.5 and exhibited good overall geometry (Table 1). The HA trimers adopt an alternating up-and-down arrangement with head-to-head interactions (Fig. 1A). Surprisingly, anomalous difference Fourier maps calculated with phases from a model showed a pronounced peak at the head-to-head interface between two neighboring HA trimers, clearly visible at the 7 σ level (Fig. S1). Fluorescence scans around the anomalous edges using the HA crystals strongly suggested that Zn^2+^ ion could contribute to the strong anomalous signal at the absorption edge near at 1.284 Å (Fig. S2). Despite the lack of Zn^2+^ in the crystallization medium, Zn^2+^ ion was tentatively refined with full occupancy and B-factor of 41–47 Å^2^ (three Zn^2+^ ions in the asymmetric unit). The divalent ion binds to the side chains of Glu68 and His137 from two adjacent trimers (Fig. 1B). In the final 2|F_o_| − |F_c_| and |F_o_| − |F_c_| difference Fourier maps, the divalent cation adopts an octahedral molecular geometry, mediated by the coordination with Glu68 and His137. They likely act as bi- and monodentate ligands, respectively, to the Zn^2+^ ion with the coordination bond lengths of 1.9–2.3 Å.

**Table 1:** Data collection and refinement statistics.

	HA-Zn^2+^ complex
**Data collection**	
Resolution range (Å)^a^	48.48–3.24 (3.36—3.24)
Space group	C 1 2 1
Cell dimensions: a, b, c (Å)	215.71 124.27 214.53
α, β, γ (°)	α = 90.00, β = 102.91, γ = 90.00
Unique reflections	87,995
Completeness (%)	99.91 (100)
I/σ(I)	7.73 (3.23)
R-pim (%)	7.89 (20.64)
Multiplicity	13.3 (12.3)
CC_1/2_	0.99 (0.92)
Wilson B factor (Å^2^)	54.83
**Refinement**	
Resolution (Å)	48.75–3.24
	(3.356–3.24)
Reflections used in refinement	87,955 (8830)
R_work_/R_free (%)_	26.3/29.5
Number of non-hydrogen atoms	24,413
Protein	23,024
Ligands	961
Solvent	428
RMS deviation (bonds, Å)	0.015
(Angles, °)	1.80
Ramachandran favored (%)	90.4
Allowed (%)	9.0
Outliers (%)	0
Average B-factors (Å^2^)	74.7
Protein	75.4
Chromophore	85.8
Solvent	16.8

^a^Values in parentheses are for the highest-resolution shell.

### Effects of divalent cations on HA proteins

**Figure 2:**
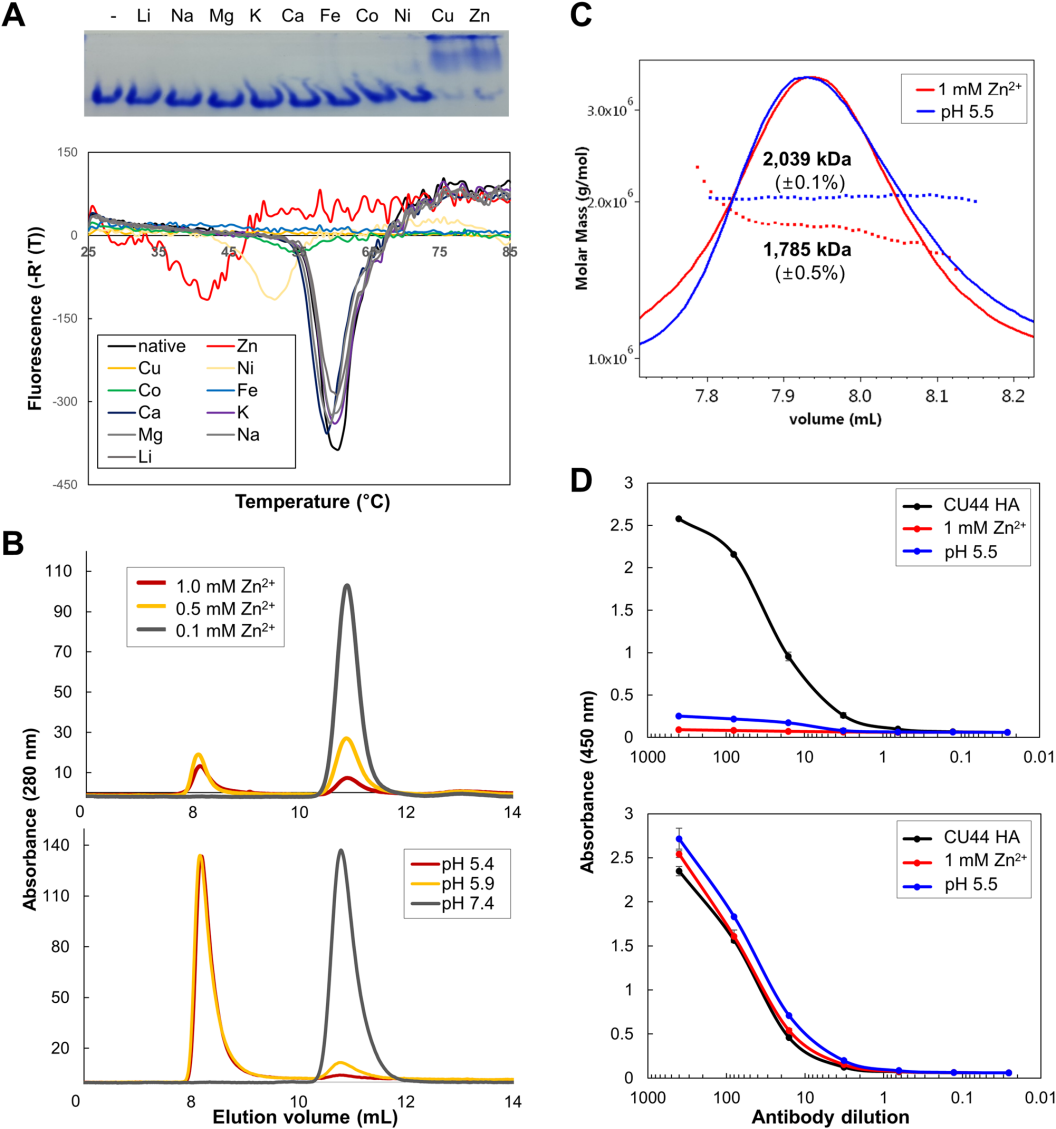
Characterization of HA proteins in the presence of divalent cations. (**A**) Native polyacrylamide gel electrophoresis results showing the band shifts in the presence of Zn^2+^ and Cu^2+^ (upper panel). The CU44 HA protein was isolated as a trimer, whereas the two cations induced multimerization or aggregation of the HA molecules. Differential scanning fluorimetry transition curves of HA proteins at different divalent cations (lower panel). HA protein was incubated at 25 °C, and then the temperature was increased by 0.5 °C every 30 s for 50 min. (**B**) The shift in elution volume of the CU44 HA in the presence of Zn^2+^ (upper panel) and acidic pH conditions (lower panel) in SEC. The experiment was performed using a Superdex 200 GL column equilibrated with 50 mM Tris (pH 8.0) and 100 mM NaCl, and the shifts were monitored as the concentration of Zn^2+^ increased from 0.1 to 1.0 mM. (**C**) The SEC-MALS results of CU44 HA. The HA was incubated with 1 mM Zn^2+^ or 100 mM MES (pH 5.5) at 24 °C for 30 min and analyzed using SEC on a Superdex 200 GL column equilibrated with PBS at a flow rate of 0.5 ml/min. (**D**) Antibody recognition of HA conformations, CR9114 (upper panel) and D2 H1-1/H3-1 (lower panel). CU44 HA was incubated with 1 mM Zn^2+^ or 100 mM MES (pH 5.5) for ELISA at 4 °C overnight. The stem-specific CR9114 or head-specific D2 H1-1/H3-1 at concentrations of 400, 80, 1, 3, 0.64, 0.128, and 0.026 ng per well was used and detected by anti-human IgG1-HRP as the secondary antibody. Absorbance at 450 nm was monitored.

In parallel, we carried out gel shift experiments involving native polyacrylamide gel electrophoresis of the CU44 HA in the presence of various divalent cations. The Cu^2+^ and Zn^2+^ cations promoted robust multimerization of HA (Fig. 2A, upper panel). To assess whether the presence of the divalent cations can impact the stability of HA, DSF was used to measure the changes in T_m_. Many divalent cation had no effect on the T_m_ values of the HA, whereas the T_m_ values in the presence of Ni^2+^, Zn^2+^ and Cu^2+^ were significantly reduced by 8 °C and 18 °C and could not be obtained, respectively (Fig. 2A, lower panel). When HA was treated with 0.1–1 mM Zn^2+^, a significant shift in the size exclusion chromatography (SEC) profile was observed. As the concentration of Zn^2+^ increased, multimeric forms of the HA were eluted at the void volume (Fig. 2B, upper panel). The HA protein sample was filtered through a 0.22 μm membrane prior to chromatography and the peak at the void volume in the SEC profile became lower as the Zn^2+^concentration increased, suggesting that larger aggregates of HA formed at high concentrations of Zn^2+^. In contrast, the HA protein also induced multimerization at acidic pH, but not to the extent of formation of the larger aggregates induced by Zn^2+^ (Fig. 2B, lower panel). Additionally, the multimerization of the HA by Zn^2+^ was not abolished by the treatment of ethylenediaminetetraacetic acid (EDTA) at concentrations of 1–10 mM (Fig. S3). The conformational changes and multimeric associations of HA induced by Zn^+2^ or acidic pH have been examined using SEC-MALS and binding of antibodies (Fig. 2C, D). The molecular weights of HA multimers induced by Zn^+2^ or acidic pH were 1785 or 2039 kDa, respectively, corresponding to the associations of approximately 9 HA trimers, and the HA multimers induced by Zn^+2^ showed a relatively polydisperse population. In addition, the conformations induced by Zn^+2^ or acidic pH were not recognized by CR9114, but by the D2 interface antibody that binds to the head domain (Fig. 2D)^29^. The results indicate that the stem conformations of HA induced by Zn^+2^ or acidic pH are not recognized by CR9114 and do not represent the native prefusion conformations. Our results thus suggest that the treatment with Zn^2+^ induces the conformations of HA, similar to those induced by acidic pH.

Comparison of the amino acid sequences of HA from different subtypes of influenza viruses revealed the conservation of the Zn^2+^ binding residues, Glu68 and His137, in H1 strains, but not in other subtypes (Table 2). In some strains, including the 1918pdm and the 1933–1936 seasonal strains, the corresponding amino acid residues of HA were substituted with Asp or Tyr, respectively. Nevertheless, the unique binding of Zn^2+^ to HA in a broad range of H1 subtype influenza A viruses reveal a subtype-specific interaction of HA with Zn^2+^.

**Table 2:** Amino acid residue variations of HA among H1 strains and subtypes.

	Residue number	
	68	137
**Strains**		
A/Brevig_Mission/1/1918	D	Y
A/Puerto Rico/8/1934	D	H
A/Albany/4835/1948	E	H
A/Malaysia/1954	E	H
A/California/45/1978	E	H
A/Victoria/36/1988	E	H
A/Texas/36/1991	E	H
A/Hong_Kong/1035/1998	E	H
A/Taiwan/141/2002	E	H
A/Thailand/CU44/2006	E	H
A/Florida/12/2007	E	H
A/Hawaii/02/2008	E	H
A/California/07/2009	E	H
A/Pennsylvania/27/2016	E	H
**Subtypes**		
H1	E	H
H2	D	V
H3	D	R
H4	D	R
H5	D	Y
H6	D	Y
H7	D	R
H8	D	A
H9	D	D
H10	D	R
H11	D	F
H12	D	N
H13	T	D
H14	D	R
H15	D	R
H16	A	D
H17	D	F

### pH-dependent conformational changes of CU44 HA

**Figure 3:**
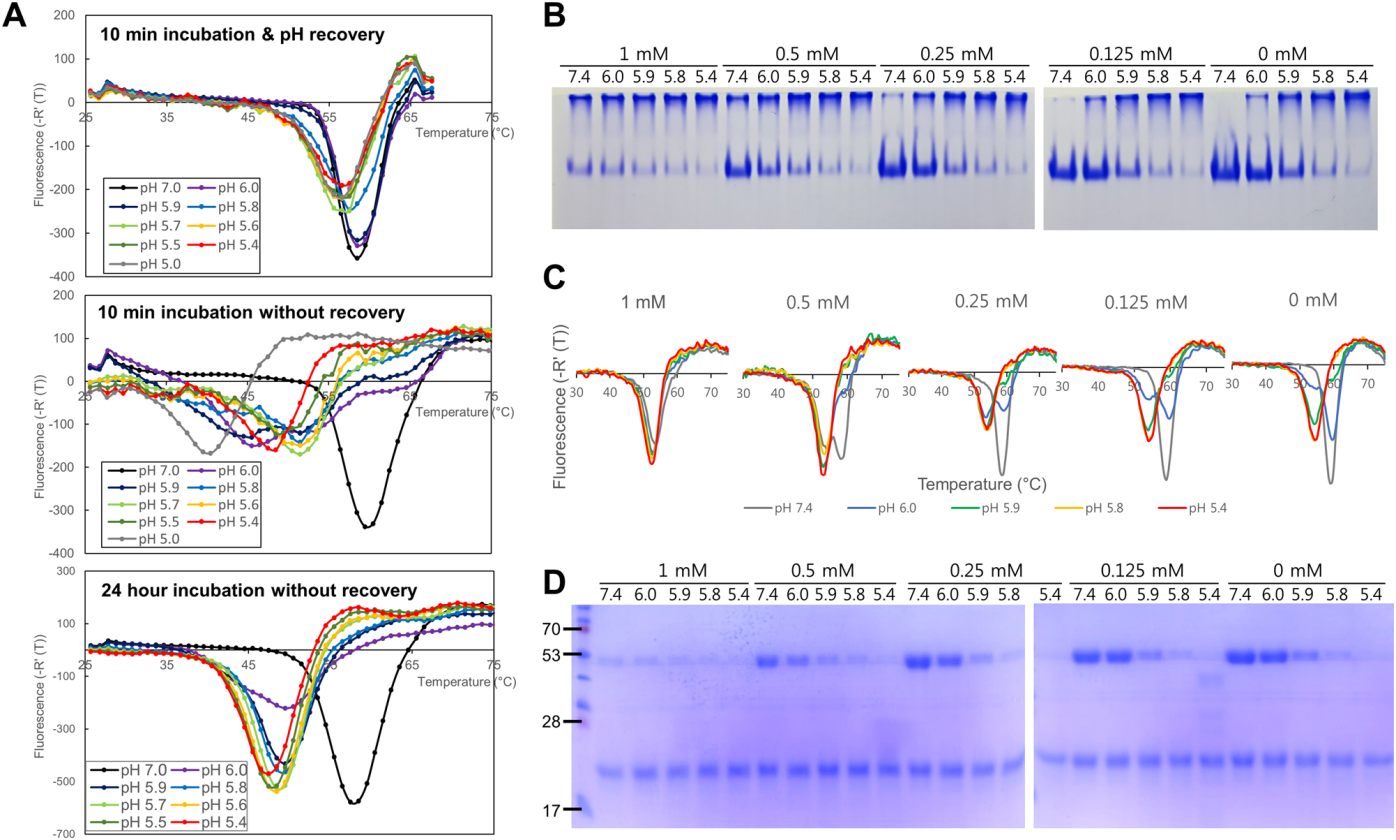
Differential scanning fluorimetry transition curves of HA proteins at different pH conditions. (**A**) The T_m_ values of the CU44 HA were measured after it was incubated at different pH from 7.0 to 5.0 for 10 min followed by rapid neutralization (upper panel) and without neutralization (middle panel) at room temperature, and 24 h incubation without neutralization (lower panel). HA protein was incubated at 25 °C, and the temperature was increased by 0.5 °C every 30 s for 50 min. (**B**) pH-dependent aggregation using native polyacrylamide gel electrophoresis, (**C**) differential scanning fluorimetry, and (**D**) Proteolytic cleavage of the CU44 HA. HA aggregated at pH 5.4 in the absence of Zn^2+^, whereas it aggregated readily at pH 7.4 in the presence of Zn^2+^, which was also dependent on the concentrations of Zn^2+^. The T_m_ values of the HA were measured at concentrations of 0, 0.125, 0.25, 0.5, and 1 mM of Zn^2+^ at different pH conditions (pH 7.4, 6.0, 5.9, 5.8, and 5.4) followed by rapid neutralization at room temperature. The T_m_ values for the CU44 HA significantly decreased from 58 to 54 °C. For the proteolytic cleavage, after the HA was mixed with 10 µg of TPCK-trypsin at 37 °C for 10 min, it was stopped by adding 5X sample buffer and heating at 95 °C for 5 min, and analysed by SDS-PAGE.

Acidic pH in the endosome induces a large conformational change of HA whose stability to an activation pH is necessary for influenza virus pathogenicity and transmissibility^6–9,14^. In order to examine the pH-dependent stability of the CU44 HA, DSF experiments were carried out by incubating the HA at different pH conditions followed by rapid neutralization. Two distinct T_m_ values were obtained for the CU44 HA, 58 °C at pH 5.9–7.0 and 56 °C at pH 5.0–5.7 (Fig. 3A, upper panel). In comparison, the conformational changes started to occur at pH 6.0 upon a 10-min incubation without neutralization, with the changes occurring at pH < 6.0 being irreversible (Fig. 3A, middle panel). The T_m_ values continuously decreased from 52 °C at pH 5.9–5.6 to ~ 40 °C at pH 5.0. Upon a 24-h incubation, two distinct conformations were apparent, with T_m_ values at 58 °C at pH 7.0 and 48 °C at pH below 6.0 (Fig. 3A, lower panel). We postulate that these two distinct conformations correspond to pre- and postfusion states of the CU44 HA and its transition from a reversible to irreversible conformations occurs at pH 5.6–5.8.

### Effects of Zn^2+^ on conformational changes and proteolytic activation of HA

To study the effect of Zn^2+^ on the pH-dependent stability of the CU44 HA, the HA protein was incubated with Zn^2+^ ion for 10 min followed by rapid neutralization at different pH conditions. Stepwise multimerization of HA proteins was observed as the pH decreased, reaching complete multimerization at below pH 5.4, whereas it occurred readily in the presence of Zn^2+^ even at neutral pH conditions (Fig. 3B). More pronounced multimerization was evident at higher concentrations of Zn^2+^. In addition, the increased concentration of Zn^2+^ reduced the T_m_ values of the HA even at neutral pH in a concentration-dependent manner (Fig. 3C). The T_m_ values decreased from 58 to 54 °C. Thus, the conformational changes of the HA induced by Zn^2+^ were similar to that occurring upon acidic incubation without neutralization, which occurred more readily as the concentration of Zn^2+^ increased.

Infectivity of influenza viruses depends on cleavage activation of the HA precursor into HA1 and HA2 by host proteases^30^. We characterized pH-dependent proteolytic activation of the CU44 HA using SDS-PAGE. The HA1 subunit was completely degraded at a pH below 5.8, as it was highly susceptible to trypsin cleavage after the acid-induced conformational change (Fig. 3D). At 1 mM Zn^2+^, the CU44 HA exhibited greatly enhanced susceptibility to proteolytic degradation even at neutral pH, similar to that induced by acidic pH.

### Effects of Zn^2+^ on HA wild-type and mutants

**Figure 4:**
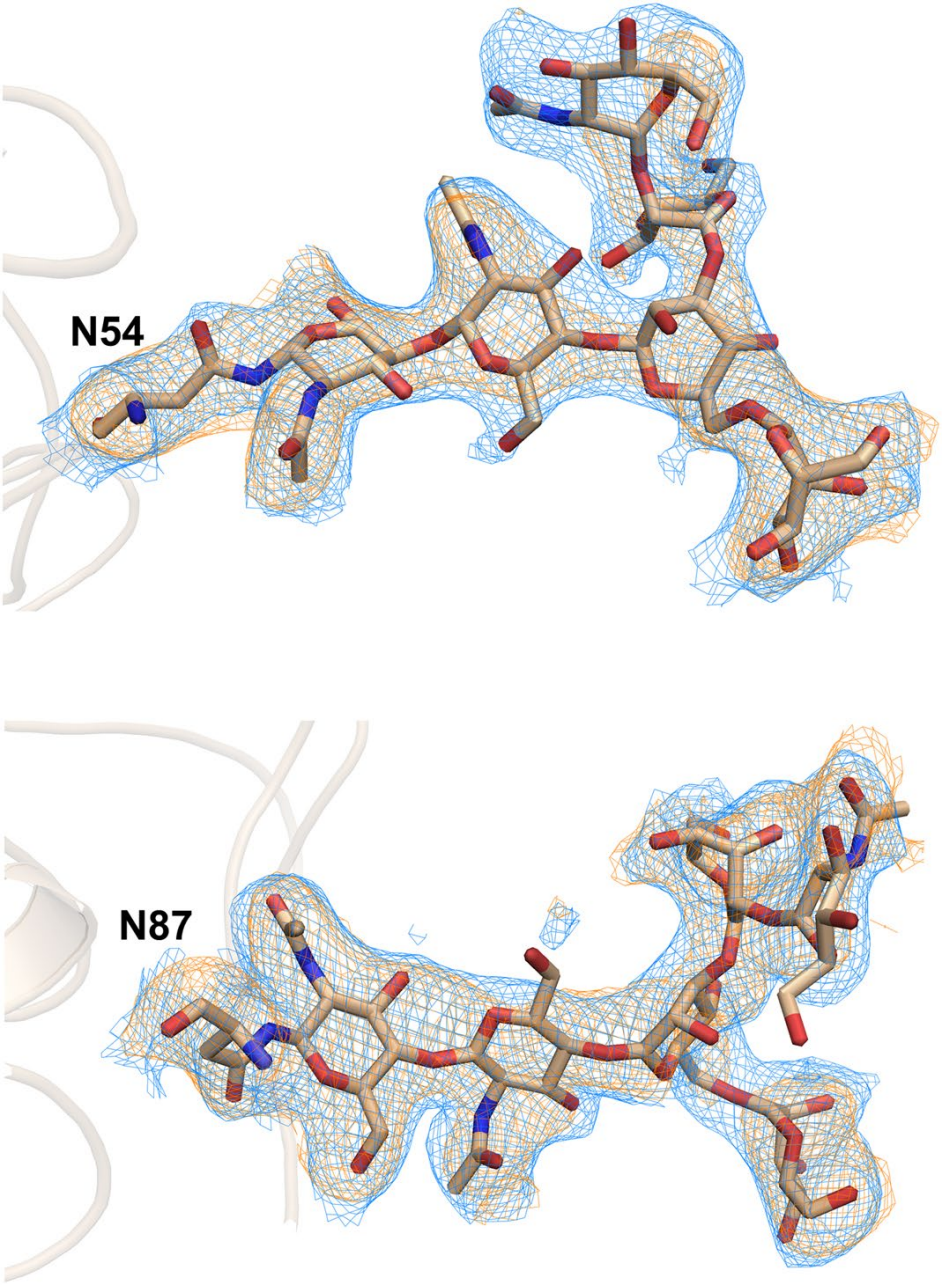
Glycan structures attached to Asn54 (upper panel) and Asn87 (lower panel) of the CU44 HA. The 2|F_o_| − |F_c_| electron density contoured at 1.0 σ and polder omit difference electron density contoured at 3.0 σ (in blue mesh) and at 3.5 σ (in brown mesh). The glycans are closer to the Zn^2+^ binding site than other glycans in the head region. The monosaccharide units are shown in stick representations. The figures were created using PyMOL (https://www.pymol.org/).

Although the thermal stability of HA was significantly reduced by Zn^2+^ and the crystal structure was determined at 3.2 Å resolution, the N-linked glycan structure was exceptionally well-defined in the 2|F_o_| − |F_c_| electron density maps, which is unusual in HA structures. It showed a branched sugar chain of up to six monosaccharide units attached to Asn residues of the head region (Figs. 4, S4). The glycans attached to Asn54 and Asn87 are much closer to the Zn^2+^ binding site than other glycans in the head region, suggesting fairly stable conformations. In addition, the multimerization of glycosylated or deglycosylated HA induced by Zn^2+^ was not prevented by the treatment of EDTA (Fig. S3, upper and middle panels).

In order to examine the effects of Zn^2+^ on biophysical properties of HA, single and double mutations at the Zn^2+^ binding site were introduced: the H137A single mutant displayed the essentially identical T_m_ of the wild-type in the absence of Zn^2+^ and the same changes in T_m_ in the presence of Zn^2+^ (Fig. S5). The incubation of Zn^2+^ with the mutants induced pH-dependent conformational changes similar to those of the wild-type, where high concentrations of Zn^2+^ equally promoted multimerization of the mutants, similar to those produced by the wild-type (Figs. S3 and S5, lower panels for the E68A/H137A and H137A mutants, respectively). Therefore, the HA, already transformed into the postfusion conformation at high concentrations of Zn^2+^, is not blocked by the mutation or EDTA treatment. When the purified HA crystallized at 10–20 mg/ml by mixing with an equal volume of precipitant solution, protein crystals were produced at less than 0.4 mM Zn^2+^, whereas precipitates were found at higher than 0.4 mM. No crystals or precipitates were found at 0 mM Zn^2+^ (data not shown). Taken together, our results suggest that Zn^2+^ binding to HA has two phases: Zn^+2^ binds specifically to the head regions of HA at low concentrations, whereas, at high concentrations, it binds nonspecifically to HA, causing conformational changes similar to those induced by acidic pH.

## Discussion

HA is a highly dynamic molecule capable of mediating a pH-dependent transition from a prefusion to postfusion state during virus entry. Induction of irreversible conformational changes upon acidification results in the formation of a stable postfusion state via a spring-loaded mechanism^31^. The prefusion conformation of HA is metastable, and the receptor binding head region acts as a clamp to keep the stem region in its metastable prefusion state at neutral pH. Acidification shifts the dynamic equilibrium of HA that favors the progression of membrane fusion^31^. We initially constructed nine mutants to improve the stability and quality of HA in solution and crystals, and the wild-type HA crystal structure derived from A/Thailand/CU44/2006 was determined as a control. Surprisingly, the highest peak in the anomalous difference electron density maps was located at the head domain at the inter-trimer interface, which was tentatively identified as Zn^2+^, based on native gel electrophoresis and absorption-edge fluorescence scan results.

The Zn^2+^ divalent cation is essential for life, although excess Zn^2+^ is toxic to the cells, possibly due to its inhibition of key enzymes^32^. Zn^2+^ is involved in many biological processes including infection of a variety of microbial and viral pathogens. During the infection, the innate immune system controls the concentration of Zn^2+^ to prevent the growth of pathogens^33^. Zn^2+^ was present in influenza virions and binds to M1 protein^20,21^. In addition, the titers of influenza viruses were significantly reduced by adding Zn^2+^ in MDCK cells^22^. The mode of inhibition by Zn^2+^ against influenza viruses was investigated in the context of DNA damage by radicals, viral protease or neuraminidase inhibition, or morphological changes of virions^21,22^. However, the molecular mechanism of divalent ion-mediated inhibition of influenza viruses is not well understood.

In the crystal structure, the HA trimers line up in an alternating up and down orientation with head-to-head interactions, where Zn^2+^ is bound between two adjacent HA trimers. This divalent cation may be acquired from the PEG 2000 used in crystallization or the medium of insect cells. In aqueous solution, Zn^2+^ can be coordinated with up to six water molecules, but it is usually tetrahedrally coordinated in zinc finger proteins and enzymes^34^. The coordination geometry of four or five coordination to Zn^2+^ is applied to catalysis with high Lewis acidity, whereas it is rarely hexacoordinated in catalytic binding sites^35,36^. In the HA structure, the carboxylic oxygen atoms of Glu68 and imidazole nitrogen atoms of His137 are directly coordinated to the divalent cation, which results in a distorted octahedral, six-coordination geometry, involving bidentate interactions of carboxylic groups. The Zn–O and Zn–N bond lengths are from 2.0 to 2.2 Å. The mean Zn–O and Zn–N distances from octahedral structures in the Cambridge Structural Database and aqueous solution are 2.10–2.11 Å^35^.

The amino acid residues, Glu68 and His137, coordinated to Zn^2+^ are highly conserved in a broad range of H1 subtypes, although they are substituted with Asp or Tyr, respectively, in the early 1918 pandemic and the seasonal 1933–1936 strains, and not conserved in other subtypes and type B (Table 2). It is likely that the Zn^2+^ binding has evolved to play a structural role in the H1 subtype viruses for the past 80 years. Moreover, it was previously suggested that the acid stability of HA during membrane fusion regulates interspecies adaptation of the influenza viruses^37^, in which the HA acid stability affects pathogenicity with pandemic capability. In this context, the arrangement of the HA trimers with head-to-head interactions in the HA-Zn^2+^ complex is unique, and the Zn^2+^-induced conformational changes of HA equivalent to those upon acidification are very striking. Recent analyses revealed that acidification and receptor binding shifts the dynamic equilibrium of HA in favor of forward progression to membrane fusion, and either receptor binding or the presence of a target membrane promotes HA2 conversion to the postfusion state^38^. Similarly, enhanced interactions of the head regions by the presence of Zn^2+^ at low concentrations can provide an efficient way of stabilization of HA, whereas high concentrations of Zn^2+^ induces significant conformational changes of HA, which may result in clustering of the HA molecules, analogous to receptor-promoted formation of the postfusion conformations.

## Methods

### Cloning and baculovirus production of HA

The recombinant HA protein derived from A/Thailand/CU44/2006 (CU44) was produced in insect cells using recombinant baculovirus expression vectors, and the vector was constructed in previous work^39^. The synthesized cDNA of the CU44 wild-type HA after RNA extraction was amplified using polymerase chain reaction (PCR). The wild-type HA gene (1-503 of HA0 based on H3 numbering) was cloned into pFastBac HT A (INVITROGEN, Carlsbad, CA, USA) downstream of the gp67 secretion signal sequence of the transfer vector pAcGP67A (BD BIOSCIENCES, Woburn, MA, USA). The cloned construct had residues 18–344 (1-326) (HA1) and 345–520 (1-176) (HA2), with a thrombin cleavage site, foldon domain, and 6 × His-tag downstream of the HA gene sequence. The recombinant HA protein contained additional plasmid-encoded residues ADPG and RSLVPR at the N- and C-terminus, respectively. Mutant HA genes were prepared by site-directed mutagenesis using a pair of complementary oligonucleotides containing the H137A mutation in the gene (MACROGEN, Seoul, Korea). Briefly, ten cycles of PCR was performed with mutant oligonucleotides to amplify the mutant HA gene, which was treated with Dpn1 (NEB, MA, USA) to remove wild type HA genes for 1 h at 37 °C. Plasmids encoding the mutant HA gene (H137A) was transformed and amplified in *Escherichia coli* strain DH5α and the recombinant bacmid was generated according to the Bac-to-Bac expression system protocol (INVITROGEN). The sequences were confirmed by automated sequencing (MACROGEN, Seoul, Korea). The CU44 HA in this study showed three mutations different from the original CU44 HA: HA1 M116I and HA2 I91V and N169S, which was used as a wild-type. These mutations also exist in nature. *Spodoptera frugiperda* insect cells were transfected with the recombinant bacmid using Cellfectin II (INVITROGEN), which were harvested at 72 h post-transfection and centrifuged at 2000 rpm for 15 min to obtain baculovirus, expressing recombinant HA in the supernatant. The transfection efficiency was confirmed by PCR after extraction of DNA from 400 μl of virus.

### HA protein purification

Baculovirus containing the CU44 HA wild-type or mutant (H137A) gene was used to infect suspension cultures of Hi5 cells, as described previously^39^. After 3 days at 28 °C, the culture medium was harvested. After centrifugation at 4000 rpm for 30 min, the supernatant was applied to a nickel–nitrilotriacetic acid (Ni–NTA) affinity column (QIAGEN, Hilden, Germany) equilibrated with 20 mM Tris–HCl, pH 8.0, and 100 mM NaCl. The column was washed with buffer containing 30 mM imidazole, and the precursor HA protein was eluted in an imidazole gradient (30–400 mM). The eluted fractions were dialyzed against 20 mM Tris–HCl (pH 8.0) and 20 mM NaCl, and hydrolyzed by TPCK-treated trypsin for 4–5 h at 4 °C for cleavage of HA0 into HA1 and HA2 and removal of the foldon and 6 × His-tag. The active form of HA was purified using Superdex 200 10/300 GL size exclusion chromatography in 50 mM Tris–HCl (pH 8.0) and 100 mM NaCl, connected to an ÄKTA FPLC system (GE Healthcare, Buckinghamshire, UK). It was concentrated in an Amicon 10,000 MWCO concentrator (Merck Millipore, Billerica, MA, USA) to 10 mg/ml for characterization and crystallization.

### Cloning, expression, and purification of Endo H

The synthesized cDNA encoding for endo-β-N-acetylglucosaminidase H (Endo H), a glycohydrolase secreted by *Streptomyces plicatus*^40^, was cloned into the PMAL-p2 plasmid and transformed into *Escherichia coli* BL21 (DE3) (INVITROGEN, Carlsbad, USA). The cells after 0.4 mM IPTG induction were harvested by centrifugation, sonicated, and centrifuged at 12,000×*g* for 20 min. The supernatant containing the Endo H was purified by affinity and size exclusion chromatography using amylose (NEB, MA, USA) and Superdex 200 10/300 (GE HEALTHCARE, Uppsala, Sweden) columns, respectively. Using the purified Endo H, HA samples were treated at 0.5 μg/ml in phosphate buffer (pH 6.8) at 4 °C for 30 h and separated from the Endo H using a 10 kDa MW cutoff membrane (MERCK MILLIPORE, Billerica, MA, USA). Deglycosylation of HA by the Endo H was determined by gel-shift assays.

### Native polyacrylamide gel electrophoresis (PAGE)

The purified proteins were prepared under native condition (mixed with 5 × loading buffer, 312.5 mM Tris–HCl (pH 6.8), 50% glycerol, and 0.05% bromophenol blue, without β-mercaptoethanol and sodium dodecyl sulfate). The HA wild-type or mutant (2–5 µg) was incubated in 100 mM HEPES or MES and 100 mM NaCl at different pH and 25 °C for 1 h. Electrophoresis was carried out in 25 mM Tris–HCl buffer (pH 8.8) and 192 mM glycine at 200 V and 80 mA for 1 h.

For a gel shift assay of divalent cations, the CU44 HA was mixed with 5 mM Li^+^, NA^+^, K^+^, Mg^2+^, Ca^2+^, Fe^2+^, Co^2+^, Ni^2+^, Cu^2+^, and Zn^2+^ ions in 20 mM Tris–HCl (pH 8.0) and 100 mM NaCl overnight. For HA-Zn^2+^ complexes, the CU44 HA was mixed with Zn^2+^ in 0–1 mM in 20 mM Tris–HCl (pH 8.0) and 100 mM NaCl overnight, which was then incubated in 100 mM MES-HEPES at pH 7.4, pH 6.0, pH 5.9, pH 5.8, or pH 5.4 at room temperature for 1 h. It was either recovered to neutral pH by mixing with 2 M Tris–HCL (pH 8.5) after 10 min, or incubated further for 24 h. The samples were submitted for native PAGE. In the case of the EDTA treatment, the HA-Zn^2+^ complex was incubated in 1 mM or 10 mM EDTA for 1 h or overnight. The electrophoresis was carried out in 25 mM Tris–HCl buffer (pH 8.8) with 192 mM glycine at 200 V and 80 mA for 1 h. The gels were stained using Coomassie Brilliant Blue R-250 (SIGMA-ALDRICH, St. Louis, MO, USA).

### Size exclusion chromatography and multi-angle light scattering (SEC-MALS) analysis

The elution shift of the CU44 HA-Zn^2+^ complex was examined by mixing the CU44 HA with 0–1 mM Zn^2+^ in 20 mM Tris–HCl (pH 8.0) and 100 mM NaCl overnight. Each sample was applied to a Superdex 200 GL column (GE HEALTHCARE) equilibrated with 50 mM Tris (pH 8.0) and 100 mM NaCl at a flow rate of 0.5 ml/min, connected to a UFLC system (SHIMADZU, Kyoto, Japan). Light scattering and refractive index were measured using in-line WYATT-787-TS miniDAWN TREOS (WYATT Technology, Santa Barbara, CA, USA), and the data were analysed by Astra 6 software.

### HA recognition by antibodies using ELISA

CU44 HA was incubated with 1 mM Zn^2+^ or 100 mM MES (pH 5.5) for ELISA at 4 °C overnight. The 8-well strip was coated with the native, Zn^2+^, or acidic-pH incubated CU44 HA in a total volume of 100 µl (2 ng/µl) in microplates (Corning). Following blocking with 1% BSA and PBS washing, primary antibodies (the stem-specific CR9114 or head-specific D2 H1-1/H3-1) were applied at 400, 80, 1, 3, 0.64, 0.128, and 0.026 ng per well at 37 °C for 1 h. They were then washed with PBS, and detected by anti-human IgG1-HRP (Abcam, Cambridge, UK) as the secondary antibody for 1 h. 150 µl of 3,3′,5,5′-tetramethylbenzidine (Sigma-Aldrich) was used as substrate and the reaction was stopped by the addition of 2 N sulfuric acid, and the absorbance at 450 nm was monitored.

### DSF

DSF was used to measure the shift in transition temperature of HA at different pH conditions, using Stratagene MX3005P (AGILENT TECHNOLOGIES, Santa Clara, CA, USA). The reaction mixture (25 µl) containing 5 µg of the HA wild-type or mutant, 5X SYPRO Orange (INVITROGEN), 100 mM HEPES or MES and 100 mM NaCl was incubated at different pHs at 25 °C for 1 h. Temperature increment at the rate of 0.5 °C every 30 s for 50 min and relative fluorescence units were recorded at the excitation and emission wavelengths of 492 nm and 610 nm, respectively. The transition temperature was calculated from the maxima of the first derivative of relative fluorescence units/temperature using MxPro QPCR Software.

### Trypsin digestion for protease susceptibility

For proteolytic cleavage analysis, the CU44 HA was mixed with Zn^2+^ in 0–1 mM in 20 mM Tris–HCl (pH 8.0) and 100 mM NaCl overnight, which was then incubated in 100 mM MES-HEPES at pH 7.4, pH 6.0, pH 5.9, pH 5.8, or pH 5.4 at room temperature for 1 h. It was recovered to neutral pH by mixing with 2 M Tris–HCL (pH 8.5) and mixed with 10 µg of TPCK-Trypsin (THERMO FISHER SCIENTIFIC, Waltham, MA, USA) at 37 °C for 10 min. Each reaction was stopped by adding 5 × sample buffer and heating at 95 °C for 5 min. Aliquots were analyzed by SDS-PAGE.

### Crystallization and data collection

The purified HA was concentrated to 10 mg/ml in 50 mM Tris–HCl (pH 8.0) and 100 mM NaCl, and crystallized by mixing with an equal volume of precipitant solution using the sitting drop vapor diffusion method at room temperature for 3–7 days. The precipitant solution contained 0.1 M Tris–HCl, pH 7.25, 20% PEG 2000 and 2% dimethylsulfoxide used as additive. After a week, small crystals were obtained. Diffraction data were collected with the crystals flash-cooled at 100 K in a stream of liquid N_2_ in the mother liquor containing 10% ethylene glycerol using synchrotron radiation sources at beamlines BL-17A at photon factory (Tsukuba, Japan) and 11C at Pohang Light Source-II (PLS-II), Korea. The crystals of HA-Zn^2+^ complex were diffracted to 3.4 Å resolution, which belong to space group C2 with unit cell dimensions of a = 215.7 Å, b = 124.2 Å, c = 214.5 Å, a = 90°, b = 102.9°, and c = 90° (Table 1). All data were processed and scaled using the XDS^41^.

### Structure solution and refinement

The crystal structure of the CU44 HA-Zn^2+^ complex was determined by molecular replacement using the CU44 HA structure (PDB ID 4EDB) as a template using PHENIX^42^. The initial solution was optimized by rigid body refinement, which produced interpretable electron density maps for the overall structure. Manual adjustment of the backbone and side chains was conducted and crystallographic refinement was carried out using the PHENIX program^42^. Difference Fourier maps, 2|F_o_| − |F_c_| and |F_o_| − |F_c_|, were used to model the active site or loop regions. After a few rounds of model rebuilding, water molecules were added using the |F_o_| − |F_c_| map peaks above 3.0 σ. The R_free_ value was used as an indicator to validate the water picking and refinement procedure and to guard against possible overfitting of the data^43^. An anomalous difference Fourier map was generated to locate the position of Zn^2+^ ion, and Polder omit maps for different regions were generated^44^. The final R factor and R_free_ were 0.263 and 0.295, respectively. Data and refinement statistics for the HA-Zn^2+^ complex are presented in Table 1. Stereochemical analysis of all refined structures using PHENIX^42^ revealed 1.6% outliers in the Ramachandran plot with 81.2% favored region among the 2577 residues. These outliers were located in the disordered loop regions.

## Supplementary information

Supplementary figures.

## Data Availability

The refined model has been deposited in the Protein Data Bank (PDB ID 6LKS).
