# Supplementary material for: Divalent cation-induced conformational changes of influenza virus hemagglutinin
Source: Sci Rep. 2020 Sep 22;10:15457. doi: 10.1038/s41598-020-72368-x (PMC7508890; doi:10.1038/s41598-020-72368-x)
Supplement: Supplementary file 1 — Supplementary figures. [file 41598_2020_72368_MOESM1_ESM.pdf]

Supplementary materials

Divalent cation-induced conformational changes of influenza virus hemagglutinin

Jong Hyeon Seok[a], Hyojin Kim[b], Dan Bi Lee[a], Jeong Suk An[a], Eun Jeong Kim[a], Ji-Hye Lee[a], Mi Sook Chung[b], and Kyung Hyun Kim[a*]

[a]Department of Biotechnology & Bioinformatics, Korea University, Sejong 30019, Korea,
[b]Department of Food and Nutrition, Duksung Women's University, Seoul 01369, Korea.

[*]Correspondence should be addressed to K.H.K.**:** Tel 82 2 3290 3444; Fax 82 2 3290 3945; Email khkim@korea.ac.kr

Keywords: influenza virus, hemagglutinin, metal ion effect, pH-dependent conformations

Contents

Supplementary Figures 1-5

Figures Legends

Fig S1. The Zn^{2+} binding site showing an octahedral coordination with Glu68 and His137 residues. The anomalous difference electron density maps are contoured at 8.0 σ in red mesh. The amino acid residues are shown in stick representation with secondary structures shown in ribbon. The figures were created using PyMOL (http://www.pymol.org/).

Fig S2. The absorption-edge fluorescence emission scan from the CU44 HA crystal on beam line 11C at PLS II. The X-ray absorption scan was performed at the zinc peak and selected energies for 3-wavelenths are shown as peak, inflection and remote points. The scan shows anomalous scattering factor real (f′) and imaginary (f″) components in different colors.

Fig. S3. Native polyacrylamide gel electrophoresis results showing the effect of Zn^{2+} on aggregation of the wild type HA (upper panel), deglycosylared (middle), and E68A/H137A mutant (lower) in a concentration-dependent manner. The treatment with EDTA did not prevent the HA aggregation. The HA wild type, deglycosylated or double mutant was incubated in 1 mM or 10 mM EDTA for 1 hr or overnight.

Fig. S4. A sideview of Zn^{2+} binding site with glycan structures attached to Asn54 and Asn87 of the CU44 HA, viewed approximately down the 3-fold axis. The glycan conformations are fold over the binding site and appears to be stabilized by binding to the head region, when Zn^{2+} is bound. The monosaccharide units are shown in stick representations in green, Zn^{2+} in magenta, and the head regions of two trimers in ribbon in different colors. The figures was created using PyMOL (http://www.pymol.org/).

Fig. S5. Characterization of the H137A mutant in the presence of divalent cations. The T_m values of the mutant were measured after it was incubated at different pH from 7.4 to 5.4 for

10 min followed by rapid neutralization at room temperature (upper panel). The mutant was incubated at 25°C, and the temperature was increased by 0.5°C every 30 s for 50 min. pH-dependent aggregation was monitored using native polyacrylamide gel electrophoresis (lower panel), in which the mutant was aggregated at pH 5.4 in the absence of Zn^{2+}, whereas it aggregated readily at pH 7.4 in the presence of Zn^{2+}, similar to the results of the wild-type.

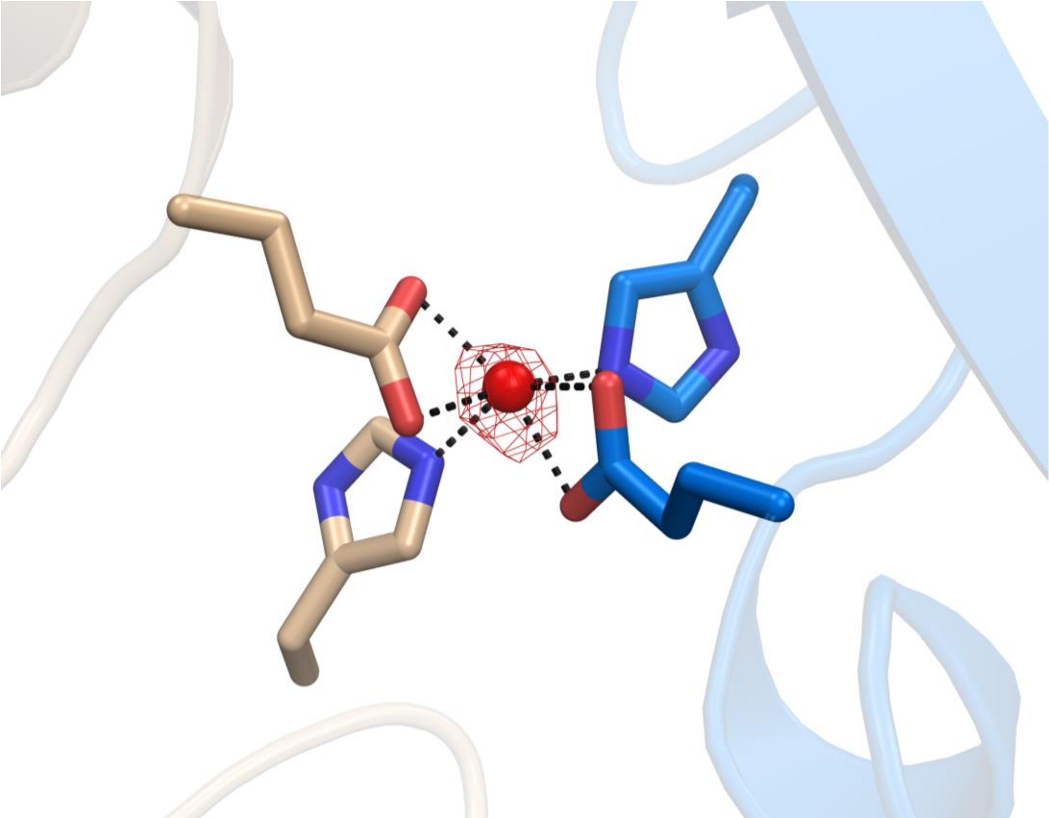

Fig. S1

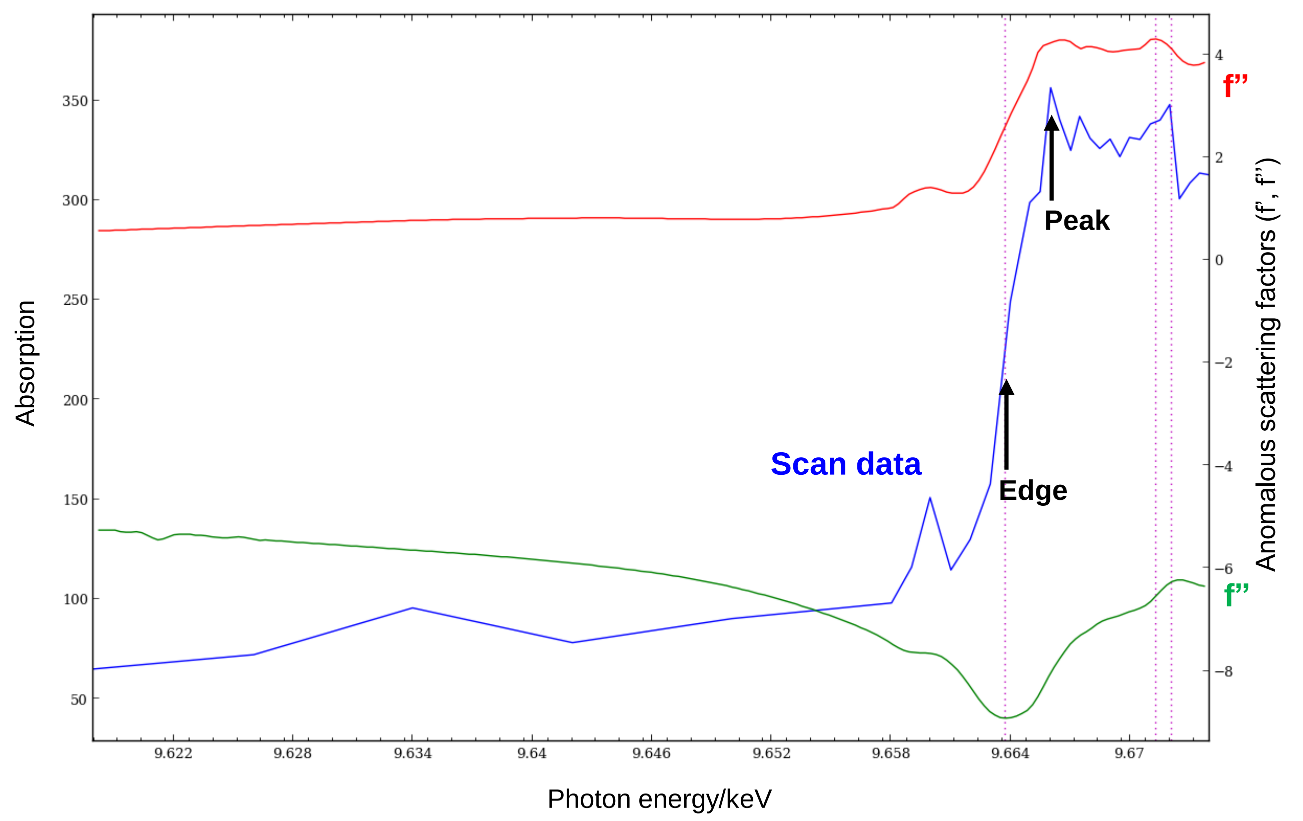

f''
Peak
Scan data
Edge
f''
Absorption
Anomalous scattering factors (f', f'')
Photon energy/keV
350
300
250
200
150
100
50
9.622
9.628
9.634
9.64
9.646
9.652
9.658
9.664
9.67
4
2
0
−2
−4
−6
−8

Fig. S2

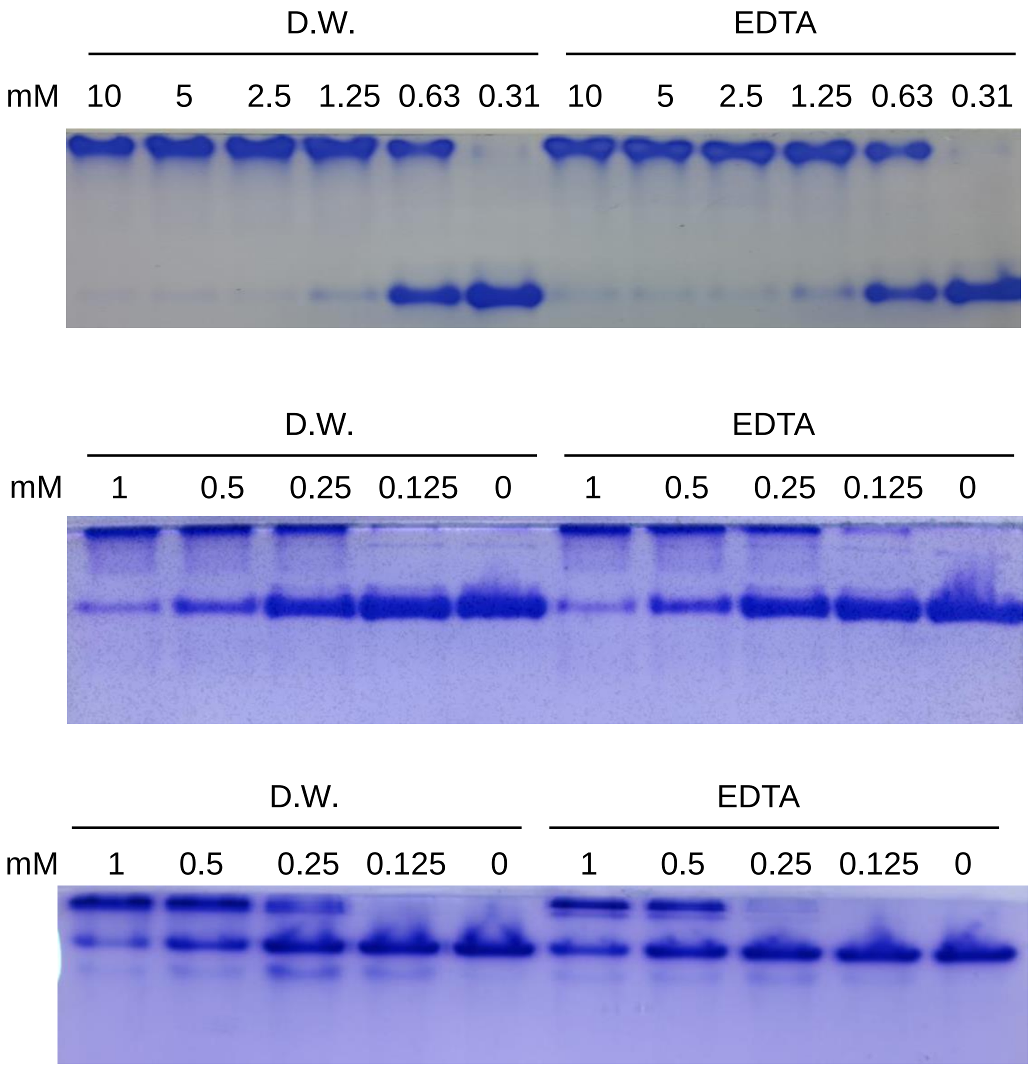
D.W.
EDTA
mM 10 5 2.5 1.25 0.63 0.31 10 5 2.5 1.25 0.63 0.31
D.W.
EDTA
mM 1 0.5 0.25 0.125 0 1 0.5 0.25 0.125 0
D.W.
EDTA
mM 1 0.5 0.25 0.125 0 1 0.5 0.25 0.125 0

Fig. S3

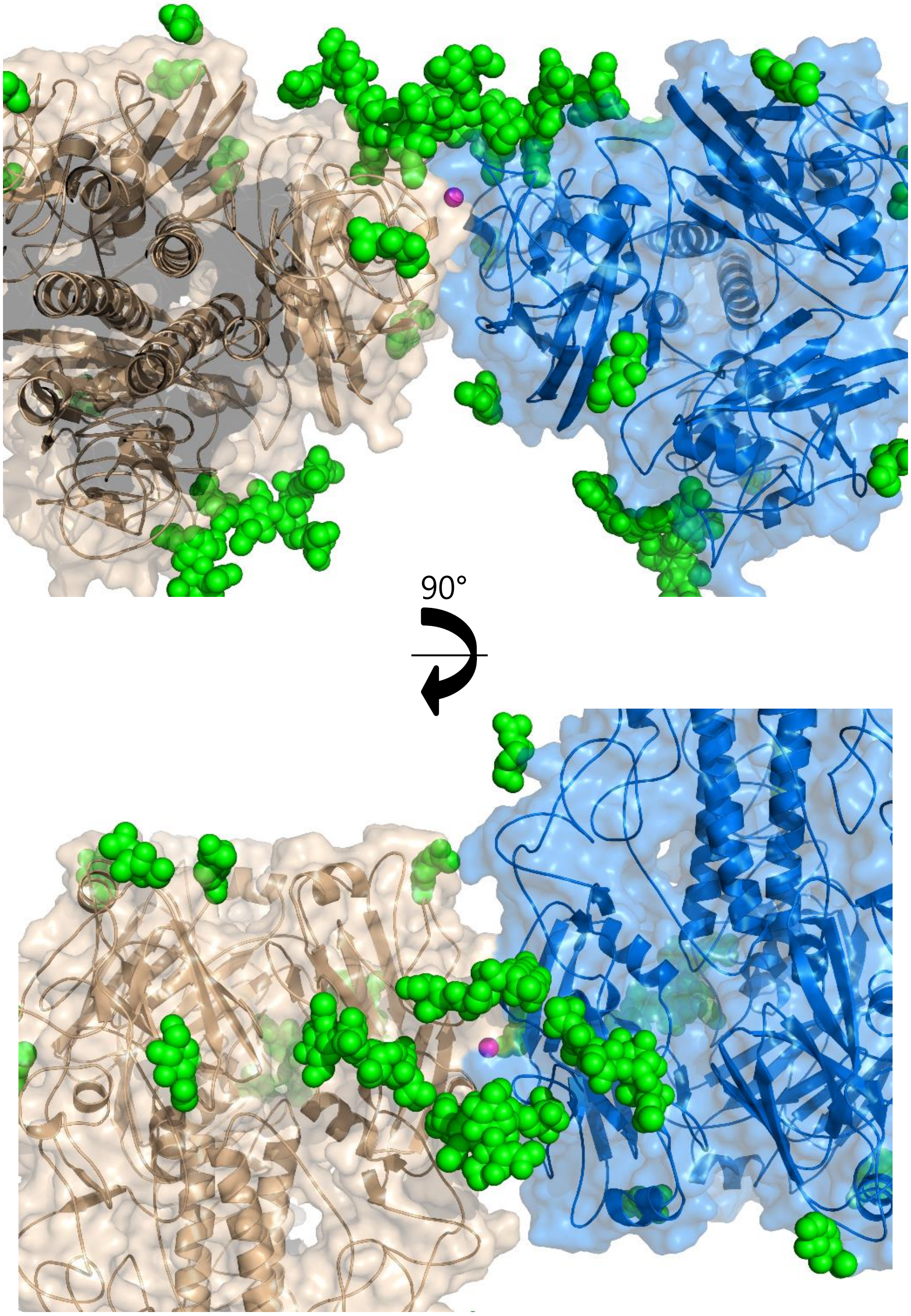

Fig. S4

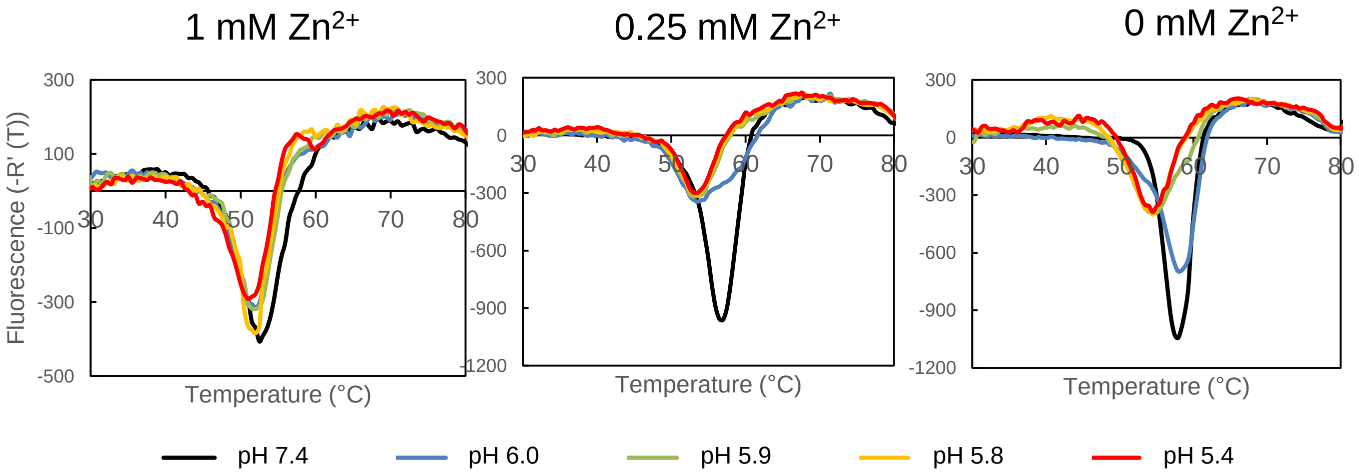
1 mM Zn2+
0.25 mM Zn2+
0 mM Zn2+
Fluorescence (-R' (T))
Temperature (°C)
Temperature (°C)
Temperature (°C)
pH 7.4
pH 6.0
pH 5.9
pH 5.8
pH 5.4

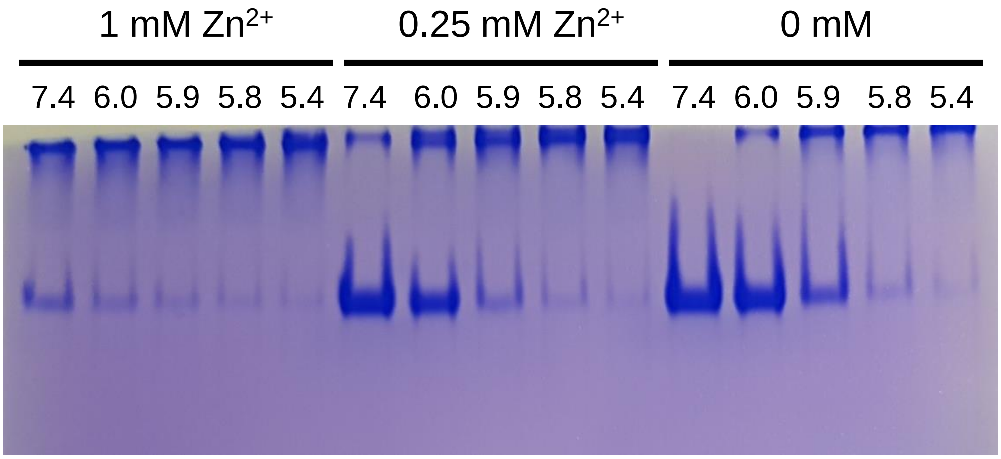
1 mM Zn2+
0.25 mM Zn2+
0 mM
7.4 6.0 5.9 5.8 5.4 7.4 6.0 5.9 5.8 5.4 7.4 6.0 5.9 5.8 5.4

Fig. S5
